# Credibility and Involvement of Social Media in Education—Recommendations for Mitigating the Negative Effects of the Pandemic among High School Students

**DOI:** 10.3390/ijerph19052767

**Published:** 2022-02-27

**Authors:** Hedviga Tkacová, Roman Králik, Miroslav Tvrdoň, Zita Jenisová, José García Martin

**Affiliations:** 1 Department of Journalism, Faculty of Arts and Letters, The Catholic University in Ruzomberok, 034 01 Ruzomberok, Slovakia; 2 Department of Russian Language, Peoples’ Friendship University of Russia (RUDN University), 117198 Moscow, Russia; roman.kralik73@gmail.com; 3 Department of Social Work and Social Sciences, Faculty of Social Sciences and Health Care, Constantine the Philosopher University in Nitra, 949 74 Nitra, Slovakia; mtvrdon@ukf.sk; 4 Department of Chemistry, Faculty of Natural Sciences and Informatics, Constantine the Philosopher University in Nitra, 949 74 Nitra, Slovakia; zjenisova@ukf.sk; 5 Department of Sociology, Faculty of Political Science and Sociology, University of Granada, 18001 Granada, Spain; jgarciamartin@ugr.es

**Keywords:** credibility, negative effects of the pandemic, online education, young generation, social media

## Abstract

In the context of considerations on the potential attenuation of the negative consequences of the COVID-19 pandemic with the use of credible social media in online education during a pandemic, the subject of our own research was the fulfillment of two goals. The main research goals were to identify, categorize, and evaluate the possibilities of using social media in online education during the pandemic from the perspective of selected teachers and students from secondary schools in Slovakia. The research methods of the first phase (qualitative) of the research involved brainstorming among nine secondary school teachers. The second research phase (quantitative) used a questionnaire, which was completed by 102 high school students from all over Slovakia. The collection of both quantitative and qualitative data was used in this research. The research results revealed the most representative opinions of teachers on the current and real possibilities of engaging credible social media in online education and the views of high school students on their desired use and involvement of social media in online education. The intersection of the two findings presents a picture of the possibilities of using credible social media in online education, which can help maintain students’ interest in online education during a pandemic. Based on these findings, it can be stated that the opinions identified in the research group of teachers correspond to a large extent with the desired use of social media in education from the perspective of students. In addition, however, students would welcome more opportunities to use and engage social media in today’s online education. The result of this research is an analysis of social media patterns applied to online education, which are of greater interest to students and could act as elements for reducing the negative consequences of the COVID-19 pandemic, i.e., six forms of online education and 24 educational activities that could contribute, inter alia, to mitigating the different negative effects of the pandemic among youth generation. The findings also benefit from the presentation of many specific options and recommendations for the use of social media in online education during a pandemic.

## 1. Introduction

In the context of the coronavirus pandemic, there are many negative consequences that are currently affecting all areas of society. In addition to the often-discussed economic crisis, or the actively-discussed healthcare crisis, there is also growing interest in the consequences of the corona crisis on the human psyche. Isolation, loneliness, a delayed return to work or school, and restrictions on social interactions are contributing to the increase in new cases of depression, suicidal ideation, attempts at self-harm, fears, anxieties, mood disturbances (elevated or irritable mood), academic procrastination, and insomnia. These and many others negative effects of the current pandemic are bringing into the issue of current public health more new patients, as well as a worsening of existing patients’ conditions [1,2,3,4,5,6,7,8,9]. The OECD states that up to one in six citizens of countries belonging to the European Union suffer from a mental health problem. In total, this includes more than 84 million people [10].

Children and young people remain the most vulnerable group in terms of considerations and recommendations to mitigate the negative effects of the pandemic on a regional and global scale [1,2,3,4,11,12,13]. Especially for today’s young people (high school and university students or young workers), a small intensification of risk factors has been seen (social anxiety, loneliness, procrastination, presence of psychotic symptoms, addiction to the internet and computer games, increased use of alcohol and psychotropic substances) [6,14,15,16]. The potential reversion of years of progress in access to education due to closures of schools worldwide is also proving to be a problem. According to the Sustainable Development Goals Report 2020, during the year 2020, as COVID-19 spread across the world, more than 190 countries had to decide to close schools; thus, 90 percent of all students across the globe (1.57 billion) were out of school [17]. On top of their social isolation, children and adolescents were also exposed to adverse social circumstances, which also affected their mental health, wellbeing, and (lack of) management of—for example—school responsibilities and tasks. We have in mind, for example, the consequences of home isolation, which may include domestic violence, psychosomatic difficulties (headaches, sleep disorders, eating disorders, etc.), or the loss of loved ones due to death [18,19,20].

Despite the fact that high school students are considered by experts as the most vulnerable group during the pandemic, experts pointed out that learning losses did not systematically increase, for example, with individual disease or as consequence of other health or economic impacts of COVID-19, but were, in fact, the outcome of remote learning [21,22,23,24,25]. In short, the shutting of education systems worldwide severed students from more than just classrooms, friends, and extracurricular activities, but also cut off the young generation from school staff members whose help and compassionate advice helped them build their self-esteem or navigate the pressures of adolescence. Thus, mental health experts “worry about the psychological toll on a younger generation that was already experiencing soaring rates of depression, anxiety and suicide before the pandemic….” [8]. This consideration was an important motive for this study. We asked ourselves, how could we set up education (and at the same time not lower educational standards) so that it is not only affordable for young people, but also attractive, inviting, and motivating for further education and personal growth? From a public health perspective, we therefore studied and presented an analysis of social media patterns applied to online education, which are of great interest to students and could act as elements to reduce the negative consequences of the COVID-19 pandemic.

Considering the potential attenuation of the negative consequences of COVID-19 with the use of credible social media in online education during a pandemic, the subject of our research was the fulfillment of two goals. The main objective of our research was the identification of (1) forms of online education and (2) educational activities carried out during the pandemic. The involvement of credible social media should have been both currently possible for online education as well as attractive from the teachers’ point of view. The research methods of the qualitative research involved brainstorming by nine secondary school teachers and a questionnaire, which was completed by 102 high school students from all over Slovakia. The results of the research present the most representative opinions of teachers and high school students from Slovakia in both the monitored areas, in order to subsequently observe any common interpretations between them. The findings conclude with a picture of the real possibilities of engaging credible social media (in the view of secondary school teachers) and the desired use and involvement of credible social media in online education, which could be helpful in relation to many negative effects of the pandemic (from the students’ view). Based on the findings, it can be stated that the real possibilities for the current use of credible social media in education, which were identified by teachers, to a large extent correspond to the desired use of social media in education from the perspective of students. In the group of students, three other possibilities for social media involvement in online education were identified, categorized, and evaluated, which from the students’ point of view could contribute to mitigating the negative effects of the COVID-19 pandemic.

## 2. Credibility and Involvement of Social Media in Education-Related Research

Social media is an innovation in Web 2.0 technology, which is the second generation of services focused on online information distribution, communication, and collaboration between users, which Web 2.0 does with audio, video, text, and images. Thus, social media includes several social networks, blogs, communities (e.g., YouTube), wikis, social virtual worlds (e.g., Second Life), and virtual game worlds (e.g., World of Warcraft). Users can use social media for a variety of activities (e.g., communicating with real-life friends, meeting new people, meeting others based on the same passions or interests, sharing content, creating images or videos, creating online content (e.g., blogging), playing online games, etc.). All of this takes place on several platforms that provide internet, including computer or smartphone devices; all of these are put into service with updates to improve peoples’ enjoyment of the social media. In addition, the use of these devices has a positive impact on their frequent use, as well as on their long-term sustainability in education [26]. It is no wonder that social media are highly attractive to today’s youth, and that they use them “whenever it suits them” [27]. The experimental question remains, and we are here primarily interested in the positive effects of the involvement of credible social media in education and the use of social media to mitigate the negative effects of the COVID-19 pandemic on the younger generation.

### 2.1. Social Media Credibility Factors

High availability, a huge amount of information, and the interest of the audience in social media content naturally also raises questions about their credibility, which is considered to be one of the main criteria for distinguishing diverse content; from fair and truthful content to that which is considered false and intentionally misleading. To remain credible, we have to decide daily which option will be better for us—or, as Kobylarek at al. pointed out, to remain credible, we must meet several conditions: (1) we must believe in the democratic process; (2) we must know what is right, and (3) we must want to choose what is right [28].

Online information has different values, and credibility, according to Wathen and Burkell, plays an important role in filtering hard-to-believe information [29], as it reminds us of the need to judge and evaluate online media, content creators (i.e., resources), and information (content) [30]. According to the so-called two-phase approach to social media credibility assessment proposed by Wathen and Burkell, (1) social media credibility factors include, in particular: (a) cognitive factors (media reliability, tradition, credibility, expertise, etc.); (b) institutional factors (social status of the media, reputation, trust in the brand, etc.); and (c) technical factors (social media design, ease of use, degree of interaction, etc.). Of course, the user’s introspective view of the social media content is also important, and therefore it is necessary to mention (2) the most important factors of credibility from the user’s point of view. In this sense, we can talk, for example, about the subjective motivation, credibility, knowledge, and social ties that an individual has within and outside social media, the current social context of the individual (e.g., online monitoring of opinion-formers and the activity that follows), their past experiences, their opinions that have been influenced by others, and the motives or purpose of the individual using social media [29].

Let us add that, in parallel with credibility, the level of information and media literacy of an individual has proven to be essential, helping the individual to identify false information or manipulative elements of online content on the Internet, where millions of different web users (professionals but especially laymen) can offer their immediate thoughts and opinions [31], whose volume in the online space is growing uncontrollably [32,33,34]. Thus, information and media literacy represent, for example, the ability of an individual to distinguish between the quantity of content (repetitive sharing in the space of social networks) and the quality of content—because in the first case, the attractiveness of the news or media dominates, while in the second, their credibility does. This distinction is not always easy to make, which is why today’s young people face the great challenge of making everyday decisions about the truthfulness of media content, in direct proportion to the amount of content that users “consume”. At the same time, experts warn us not only about the increase in the volume of fake media content [35]—despite the fact that social media are considered the most-used source for scientific information [36]—but also about information overload [37], which leads to inattention [37,38,39,40], an impaired ability to decide on the truthfulness of content [41], and to a limited attention span [42].

### 2.2. Involvement of Credible Social Media in Education to Reduce the Negative Consequences of the COVID-19 Pandemic

Although experts see the positives of social media, related research has tended to emphasize the risks and primarily negative effects of the use of media on the younger generation; for example, the negative consequences of excessive media use may contribute to mood and anxiety disorders [43], negative feelings regarding school work, appearance, family, friends, the attended school, or life [44], gender roles, sexual relationships, body image disturbances, obesity, and so on. On the other hand, among researchers, we also find ambitious attitudes towards social media and their use among young people today [45,46,47]. Finally, there are several positive effects of social media on young people; for example, social media influences perceptions of social support, stress, and wellbeing [48], may lead to higher social capital in schools and online relationships [49,50], health and health communications [51], raise awareness on important issues [52], and lead to higher social capital in relationship to self-esteem [46,53], subjective wellbeing [50], or even procrastination [54,55].

Interestingly, experts disagree on the impact of social media on student wellbeing, although most (especially in older research) cite negative effects of the COVID-19 pandemic associated with the use of social media [56,57,58,59,60,61,62,63,64,65,66,67,68,69,70]. According to Meier, this is because “most prior research on the effects of smartphone devices and social media on well-being has worked from either the” technology addiction “or” screen time “approach” [68].

In the context of youth education, social media can be seen as diverse and, at the same time, attractive tools, inviting students to participate in collective education (as opposed to autonomous learning). Online networking of students from different countries is proving effective, for example, through joint educational projects or environmental initiatives. Mutual enrichment brings not only a sense of reciprocity (as opposed to isolation), but also the creation of joint projects, the perception of (cultural) differences, the development of language skills, etc. Active forms of involvement, implementation, and use of social media in education subsequently has not only an educational character, but also a character which develops individually and within teams, and brings positive incentives that can contribute, inter alia, to mitigate the negative effects of the pandemic on the younger generation. From a public health perspective, we therefore studied and presented an analysis of social media patterns applied to online education, which are of greater interest to students and could act as elements to reduce the negative consequences of the COVID-19 pandemic such as depression, suicidal ideation, attempts at self-harm, fears, anxieties, mood disturbances (elevated or irritable mood), academic procrastination, and insomnia—bringing more new patients as well as a worsening of existing patients in public health systems. We also thought about other social circumstances that affect young people, for example, the loss of loved ones due to death and the consequences of home isolation—which includes domestic violence and psychosomatic difficulties (headaches, sleep disorders, eating disorders, etc.) [18,19,20].

## 3. Methodology

This research consisted of two phases; i.e., qualitative and quantitative.

The main goal of the research was to identify, categorize, and evaluate the possibilities of using social media in current online education in which:

(1) Secondary school teachers see current opportunities for the positive involvement of credible social media in education; and, simultaneously,
(2) High school students see the use of social media as a tool for maintaining their own interest in online education.

The collection of both qualitative data (research on secondary school teachers) and quantitative data (research on high school students) was used in this research.

The results of this research will present the mutual intersection of the two monitored areas to present a picture of currently possible and yet attractive online learning opportunities (i.e., in the teachers’ view), and at the same time the desired use of social media as motivational tools to maintain interest in online education (i.e., in the high school students’ view). This study presents an analysis of social media patterns applied to online education, which are of great interest to students and could act as elements in the reduction of the negative consequences of the COVID-19 pandemic.

### 3.1. Qualitative Research Procedure

#### 3.1.1. Design of the Qualitative Research

The main objective of our research was the identification of (1) current forms of online education and (2) educational activities carried out during the pandemic. The involvement of credible social media should have been both:

(a) currently possible in online education;
(b) attractive from the teachers’ point of view.

A total of 9 secondary school teachers were contacted for qualitative research. All respondents were investigated together with the use of an online brainstorming method. First of all, teachers were asked to identify the main forms of current online education. Secondly, they tried to identify educational activities implemented for online education during the pandemic. The result of the online meeting was a list of the major forms of current online education and a list of major online activities.

In order to get an even more accurate picture of the online activities carried out during the pandemic in the surveyed schools, we asked teachers for an evaluation of the list of educational activities. Each teacher rated each activity they had actually carried out and how attractive they considered it. Zero points were reported for activities that teachers did not implement and/or did not find attractive. The maximum number of points obtained for each activity was 9 points. The result was a “number of consents” listed for each of the educational activities among the nine respondents.

Identified categories (i.e., forms of online education) and variables (i.e., educational activities) were transferred to the qualitative questionnaire on an ad hoc basis, thus being prepared for further quantitative evaluation among high school students (i.e., in the 2nd research phase).

#### 3.1.2. Participants

The qualitative phase took place in a group of nine secondary school teachers of both genders with a minimum of six years of experience. From the point of view of the categorization of pedagogical staff responsible for the implementation of the school educational program in the Slovak Republic, all respondents to this part of the research (i.e., the 1st research phase) were fully qualified teachers; according to § 29 Act no. 138/2019 Coll. (i.e., the Act on Pedagogical Employees and Professional Employees), included in the “career level ‘independent pedagogical employee’” with either the first or second attestation [71].

#### 3.1.3. Research Method

The research methods for the 1st research phase involved brainstorming, a method that can be used to generate ideas for solving clearly defined problems or for searching for the most effective solutions. Qualitative methods typically have three basic steps: (1) idea capturing, with a focus on the quantity of ideas; (2) group discussion based on an atmosphere without judgment; (3) the selection of ideas, based on the combination and improvement of main ideas [72,73,74]. According to Alex Osborn, who is the author of both the brainstorming method and its methodology, we not only gather our own ideas during this method, but also consider and build on the ideas of colleagues, thus covering the problem from all possible angles [75]. Thus, this method uses collective thinking, which is aimed at various potential solutions; that is why it other terms used for brainstorming include “insight” or “mind mapping”. According to experts, the implementation of group brainstorming allows you to bring together discussants and develop some good and deep ideas [76,77,78,79], because this technique “encourages distant individuals to participate and puts everyone on the same pitch” [80].

In our research, brainstorming took place through the Zoom application (NASDAQ, San Jose, California, USA) in September 2021 and we considered it, despite its mostly online form, as beneficial. Ultimately, many experts also consider online brainstorming as “the most realistic solution for brainstorming meetings”, even in a university environment [81]. Interesting research has been carried out, for example, by Barki and Pinsonneault, who provided a closer look at EBS brainstorming groups in terms of idea quality. The authors pointed out that the quality of ideas generated by small groups using four brainstorming technologies (nominal, verbal, EBS-anonymous, and EBS-non-anonymous) seem to be equally effective, i.e., there was no statistical differences in terms of idea quality [82]. Moreover, Gallupe et al. provided two concurrent experiments conducted with groups of varying sizes. Based on this research, the larger research groups “generated more unique ideas and more high-quality ideas, and members were more satisfied when they used electronic brainstorming than when they used verbal brainstorming.” Authors interpret these results “as showing that electronic brainstorming reduces the effects of production blocking and evaluation apprehension on group performance—particularly for large groups” [83]. Further similar findings could be discussed, but this is not our goal. However, we point out that the electronic form of brainstorming is an effective research method that brings about many significant and effective solutions.

### 3.2. Quantitative Research Procedure

#### 3.2.1. Design of the Quantitative Research

The quantitative phase had one research question: which of the current possibilities for the involvement of social media in education do the researched high school students see as a tool for maintaining their own interest in online education?

The evaluation took place through a questionnaire of a group of 102 high school students. The results represent an analysis of social media patterns applied to online education, which are of great interest to students and could act as elements for reducing the negative consequences of the COVID-19 pandemic.

#### 3.2.2. Participants

The 2nd research phase took place in the time interval October–November 2021. This research phase continued with the distribution of a questionnaire completed by 102 high school students from several secondary schools throughout Slovakia, a small country in the east of Europe with a population of over 5.4 million.

Data collection was performed via so-called single-stage clustered sampling. This is a “once-time sampling method in which the researcher creates several groups of people from a population where homogeneous characteristics are representative and have the same chance of being part of the sample” [84]. During the research, we worked with several small groups of 3rd and 4th grade high school students. The research sample of high school students consisted of students from western, eastern, southern, and northern Slovakia. Despite the fact that this research is not representative, we have tried to address the widest possible geographical area, in which we were relatively successful. The clustered sampling method allowed us to combine high school classes from all over the country into one cluster (*n* = 102), which then “defined the entire population” [84] of high school students from the 3rd and 4th years in Slovakia.

#### 3.2.3. Research Method

We chose the research method questionnaire because of the reliability of the evaluation of the findings and because of the efficiency of the online distribution of the questionnaire at the time of the third wave of the pandemic in Slovakia.

The questionnaire consisted of 29 questions, which were formulated on the basis of the findings of the first part of the research, i.e., in the framework of brainstorming, which identified six categories and 24 variables (i.e., educational activities). The researched students had the possibility of also identifying other uses for social media in online education in an open-ended manner.

The selection and contacting of research participants took place with the use of teachers who were involved in the research in its first qualitative phase, and who were directly involved in the data collection through an online questionnaire. To avoid any problems with misunderstandings, a questionnaire was given to 7 students for their comments during the trial phase (i.e., in the process of creating and formulating the questions). Young people had to express whether the questionnaire was understandable to them, where they were not sure whether they understood the question correctly, and so on. There was one ambiguity in the trial phase, which was resolved immediately in the questionnaire.

The quantitative questionnaire contained several areas, including six research categories: (a) Demographic questions (questions 1–3); (b) Questions concerning year of education and type of study (questions 4–5); and (c) Questions examining respondents’ subjective views on the use of social media as motivational tools to maintain one’s interest in online education (questions 6–29).

The students’ task was to evaluate each of the research variables as “I consider attractive” (1 point) or “I do not consider attractive” (0 points). In order to avoid possible ambiguities in the meaning of the items, each research variable also contained two specific examples if this proved necessary during the comprehension check of the questionnaire provided to the first 7 high school students (i.e., during the pre-phase). In the questionnaire was, for example, these examples: The variable “interactive online quizzes” gave the example of “web Kahoot”, or the variable “educational online games” gave the example of “wolves” (in Slovak), which were already well-known to high school students.

## 4. Results

The results of this research represented the most representative opinions of the secondary school teachers and the high school students from Slovakia in two research areas—the intersection and common interpretation of which represents the main findings and benefits of the research.

### 4.1. Identification of the Currently Possible as Well as Attractive Uses of Credible Social Media in Education—The View of Teachers

The aim of the first phase of the research was to identify the possibilities for the involvement of credible social media in online education during the pandemic. The involvement of credible social media should be both currently possible for online education as well as attractive for students.

The research results resulted in a final six categories and 24 variables discussed by the teachers. Most of the surveyed teachers agreed with the accepted findings from the brainstorming (*n* = 9). Our findings, including the number of teachers who agreed with the examined variables, are shown in Table 1. Each teacher rated every activity they had actually carried out and which they also considered attractive. Zero points were reported for activities that the teacher did not implement and/or did not find attractive. The maximum number of points obtained for each activity was nine points (i.e., 100%).

As we see in Table 1, within the “information learning” category, teachers defined the following four variables in our research set: *(1) Poster creation; (2) creation of video presentations and short films; (3) creation of thought maps; and (4) creation of cartoons and comics.* These are activities that, according to teachers, develop the student’s “creative work with information”, helping to build skills such as the ability to find information, apply information on a given topic, or distinguish ways to accomplish a task and overcome the initial problem of an initial lack of information on the topic.

Collaborative education can also be understood as interactive and at the same time group or team education. Under this name, the teachers of our research group also identified four variables. Under the variable (1) *interactive online quizzes, teachers used the Kahoot website* (https://kahoot.com/ 17 November 2021) during the pandemic, LoQuiz (https://loquiz.com/ 17 November 2021) was among the interactive content that allowed them to create online competitions and teacher games. In the framework of (2) *online lectures and courses of partner educational institutions*, tasks aimed at developing critical thinking were used during the pandemic among teachers (for example Train Brain, https://www.trainbra.in/sk/ 17 November 2021), websites about personal development and careers (for example What I do (in Slovak), http://www.corobim.sk/ 17 November 2021), and even educational online games (for example wolves (in Slovak), https://vlcata.sk/ 17 November 2021). Teachers also talked about the attractive opportunities that (3) *online lectures and courses provide within the YouTube channel*. Courses provided by foreign universities (for example Udemy, https://www.udemy.com/ 17 November 2021), by lay people (for example Skill Share, https://www.skillshare.com/ 17 November 2021), and by Slovak experts (for example History Otherwise (in Slovak), https://www.youtube.com/channel/UCtW6UE6h-Na5E5BCLBafbcQ 17 November 2021 or Get Wise (in Slovak), https://www.youtube.com/channel/UCA0qaLDDrr3m4MUQ0L_cFzg 17 November 2021), as well as virtual tours of museums and their exhibits (for example the British Museum YouTube channel https://www.youtube.com/user/britishmuseum 17 November 2021). The last, and therefore the fourth, variable was (4) *project teaching, which focused on working in pairs or smaller groups*. Teachers emphasized the attractiveness of an interactive group learning process, which “uses an attractive form of learning using social media and cooperation between students.” Teachers perceived collaborative forms of education as attractive to students because “students could gain new knowledge, values, but also experience (albeit mediated) as a result of solving problems that other people had to deal with in the real world.” Subsequently, however, the high school students themselves were invited to get involved in the topic and share their views, experiences or attitudes, etc.—e.g., actively participate in the educational process.

The category “technology skills training” included the following variables: (1) *Information retrieval* (for example, education focused on world wide web search services, multimedia encyclopedias, databases, etc.); (2) *use of communication tools* (for example, opportunities for online cooperation in domestic and international contexts, opportunities for information sharing (Zoom, MS Teams), etc.); (3) *productive online tools* (for example, training focused on working with graphic editors, presentation programs (Dropbox, MyHomework), etc.); and (4) *graphic online tools* (for example visually appealing posters, Adobe Photoshop, GIMP, online tools focused on graphic design such as Canva, Freepik, Pixabay, etc.). In our research, this is a category that, although it sounded important in terms of the skills currently necessary for the younger generation, the researched teachers doubted its attractiveness. Teachers also saw the lack of education focusing on the development of technological skills in the curriculum of non-technical subjects in Slovak schools as a problem.

Within the category “education using social networks”, the surveyed teachers also defined four variables: (1) *Information from educational institutions* such as libraries, museums, galleries, etc., that teachers used in teaching via their Facebook social sites (e.g., Hermitage Museum in St. Petersburg, Russia; Van Gogh Museum in Amsterdam, Netherlands; Vatican Museums in Rome, Italy, etc.). Within the social network Facebook, teachers have also identified the second research variable, which is (2) *Messenger as a communication channel for experiential learning activities and group projects*. During the pandemic, according to teachers, Messenger became a tool with several benefits: students stayed in touch, and those who were absent from teaching could follow the development of the tasks or goals of class projects; Messenger also proved to be a tool for coordinating tasks, with information being visible to everyone in the group at the same time (unlike, for example, e-mail communication) and so on. The third variable (3) *own student research through a questionnaire* is related to the social network Twitter, in which several teachers initiated several ideas for student research. Finally, (4) *the official websites of cultural institutions* were among the variables. During the pandemic, teachers in online education often used free online admission, for example, to The Hermitage Museum (https://bit.ly/33nCpQg 19 November 2021), The Metropolitan Opera—which provided free high-definition live broadcasts (https://bit.ly/2TTSr1f 19 November 2021)—or to the Vatican Museums and the Sistine Chapel (http://www.vatican.va/various/cappelle/sistina_vr/index.html 19 November 2021).

The fifth research category, named “active self-education (constructivism)”, contained the following four variables: (1) *Project teaching (individual)*; (2) *education using podcasts*; (3) *education using brainstorming and group reflection;* and (4) *education with an emphasis on practical (individual and group) learning* (i.e., learning by doing). According to teachers, all variables were activities that were based on two assumptions: independent and collective education. Both assumptions are closely related to the theory of constructivism, which in addition to art (a movement belonging to the first wave of the avant-garde of the 20th century) or psychology (the basis of processes being individual activity) is also present in education, based on Piaget’s theory of child development. The Constructivist theory of learning states that man is an active factor in the process of cognition and that his knowledge, skills, or experience grow with each additional piece of information or experience. Thus, individuals can develop and increase their knowledge and skills themselves, using the people around them (e.g., teachers) or various tools (e.g., social media). Social media is well established in this theory, as it not only enables the development of knowledge and skills, but also a wide range of interaction processes—for example, in project or group teaching, which the researched teachers appreciated and used. In addition, according to teachers, active self-education using social media is not only reflected in group work, but also in “individual tasks that have a theme and do not specify how it will be implemented.” The same topic is then presented in the class group using various methods and ways that have been chosen by the students. According to teachers, this “creates not only a diverse perception of reality among students, but also an experience of how to capture this reality, present it to classmates or apply it in life.” The use of social media in the implementation of the assigned tasks appears to be a “deep well of possibilities” from which each student “chooses what he is inclined to do, what entertains him or is a current challenge.” Teachers believe that the results of such a teaching process is knowledge that students acquire actively and with greater understanding.

Within the last category, “relationship-oriented education”, teachers identified the following variables: (1) Community education and social assistance; (2) experiential learning (within indoor activities); (3) synchronous (online) communication; and (4) online workshops. Within the first variable, community education and social assistance, teachers had in mind mutual assistance between students during online education, as well as assistance outside the classroom. In the first case, they mentioned—for example—tutoring (via zoom or messenger), but also joint online education, when at most two classmates shared one computer in one of their households (the reason for this being the lack of computers in families with several school-age children). In the second case, the first variable “community education and social assistance” consisted of social activities that occurred “outside” the classroom (for example, sorting and collecting paper, making a gift for a senior from Social Home for Seniors, etc.). Other findings related to teachers’ experiences of experiential (indoor) learning, which also contributes to building informal relationships in education. Teachers talked about online versions of various simulation games and role-plays, as well as dramatization and short theatrical productions, which they tried during the pandemic. Their experiences were profound: “Experiential learning evokes a lot of positive emotions among students, which can be seen during education in the real world (sports activities, outdoor activities, etc.) and online (artistic activities, activities aimed at personal growth, etc.)”. This suggests that even during online learning, experiential learning not only creates intensive learning and positive feelings from one’s own experiences, but is also an attractive alternative to traditional education, which emphasizes the interpretation of the curriculum and memorization of information. Teachers also state that in many secondary schools in Slovakia, the number of online workshops is also growing during the use of online education. Teachers state that, for example, team mind mapping took place during their implementation of online classes; students were asked to “think differently”—together they solved a problem, etc. The workshops met with interest from students, while teachers praised the fact that “teachers and students were partially and sometimes even fully involved in joint activities, which again strengthened relationship behavior.” Finally, with the use of social media during the pandemic, teachers also appreciated the positives of “synchronous (online) communication”. According to the researched teachers, this research variable brought students a number of benefits (immediate communication and feedback, synchronous connection of the student with the discussion, etc.). Blaho and Karvaš even stated that “it is the Internet that has significantly strengthened the social aspects of education by enabling users to communicate synchronously with each other” [85]; i.e., the virtual world with its possibilities (in a sense, paradoxically and unexpectedly) also contributes to online education with an emphasis on relationships. However, teachers talked during brainstorming about social media in the context of synchronous communication, such as for connecting people or their use as tools for finding new information and contexts. However, according to teachers, this significant characteristic of social media does not diminish the importance of social media as a space for improving the relationships between teachers and students.

### 4.2. Identification and Evaluation of Opportunities for the Involvement of Credible Social Media in Education—High School Students

The second phase of the research aimed to evaluate forms of online education (across six categories) and, if necessary, to identify other desired uses for and ways of involving credible social media in online education. Based on this goal, we asked which of the current possibilities for the involvement of social media in education do researched high school students see as a tool for maintaining their own interest in online education?

The results represent forms of online education that could use social media to help maintain the interest of high school students in Slovakia in online education during the pandemic, and thus act as elements to reduce the negative consequences of the COVID-19 pandemic.

Through a questionnaire, 102 students evaluated each of the research variables as either an attractive learning activity (1 point) or an unattractive learning activity (0 points), i.e., the maximum positive result was 102 points. The results represent forms of online education using social media, ranked from most attractive to least attractive, and are the answer to one of the research questions within the quantitative research phase.

As part of the questionnaire, students were given the option of a “different (own) answer” in each of the six categories examined. This possibility brought several other interesting findings. The presentation of the findings in the group of high school students is shown in Table 2.

Through a questionnaire, 102 students evaluated six predetermined categories and, within them, 24 variables proposed by nine teachers during the first, i.e., qualitative phase of the research. As we can see in Table 2, the most attractive forms of online education during a pandemic are considered by the respondents to be the following: (1) Collaborative education (recorded by 91 students 89.18%); (2) active self-education—constructivism (recorded in 89 students, 87.22%); (3) education using social networks (recorded in 78 students, 76.44%) and (4) education with an emphasis on relationship behavior (recorded in 76 students, 74.48%). From the students’ point of view, information education (recorded in 11 students, 10.78%), and even more so education focusing on the development of technological skills (recorded in 8 students, 7.84%), is significantly less attractive.

In the open-ended questions of the questionnaire, students were also willing to give examples of their own ideas for using social media during online education during a pandemic that they find attractive. Based on their categorization, the two most common opinions were defined:

(1) The other desires of the students were that social media in online education during a pandemic should also be more attractive to the individual approaches of teachers to their students. This opinion represented answers that pointed to “teacher’s conversations with the student outside the group’s view, “or, for example, the student’s desire for the teacher to “use the messenger more often for personal communication with the student” (this type of answer was recorded in 40 students—39.2%).
(2) According to students, social media in online education during a pandemic should also be used with a greater involvement of smartphone educational applications in subjects in online education. Research students have relatively specific ideas about how this would be possible; for example, during online education, students suggested the use of smartphone applications that develop language skills, help with 3D graphics, or even make “boring subjects like chemistry or physics” fun (this type of answer was recorded in 34 students—33.32%).

### 4.3. Common Interpretation of Research Findings

As we see in Table 3, students rated the following learning activities (variables) most positively: *Online lectures and courses within the YouTube channel* (recorded in 35 students—34.3%; Collaborative learning); *education with an emphasis on practical learning, i.e., learning by doing* (recorded in 33 students—32.34%; Active self-education); *project teaching—work in pairs or smaller groups* (recorded in 32 students—31.36%; Collaborative education); *obtaining information from educational institutions within the social network Facebook* (recorded in 30 students—29.4%; Education using social networks); and *experiential learning within indoor activities* (recorded in 26 students—25.48%; Education with an emphasis on relationship behavior).

**Figure 1:**
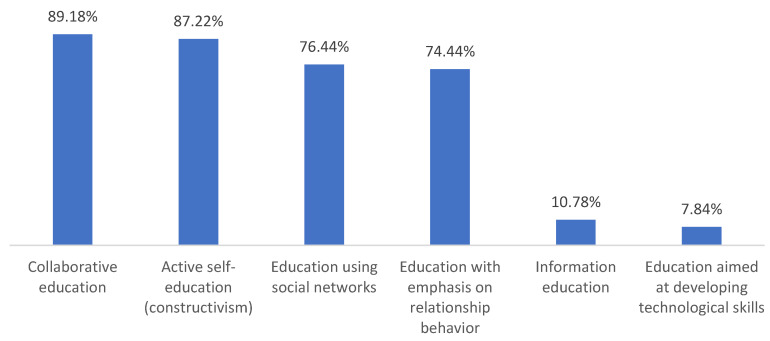
The intersection of research findings—educational activities (research variables).

The intersection of research findings on educational activities (research variables) is provided with the use of the following bar chart—see Figure 1.

Based on the findings, it can be stated that (1) the real use of credible social media in education during the pandemic identified by teachers in the first phase of the research was met with a positive response from students whose task was to (2) evaluate six forms of online education and 24 educational activities as part of online education during a pandemic. In particular, a more pronounced positive evaluation of four categories can be seen, i.e., forms of online education during a pandemic in a research group of high school students.

## 5. Discussion

Synonyms of the term credibility, such as the words trustworthiness, trust, credibility, reliability, or truthfulness [86], suggest that credibility is the basis of an individual’s qualitative relationship to information, to the online content creator, to online media, and to technology itself. Therefore, not only during the corona crisis, youth education, in which social media is involved and used, must respond effectively to the amount of online content that carries real and truthful, but also seemingly true, misleading, or even false, content [33,34,35,36,40,87,88]. The situation is not helped by the risk factors present (social anxiety, loneliness, procrastination, presence of psychotic symptoms, etc.) or unfavorable social circumstances [20] (home isolation, domestic violence, psychosomatic difficulties), which cause mental difficulties, discomfort, and failure to manage school duties among today’s young people [1,2,3,11,12]. At the same time, young people are exposed to these negatives to a much greater extent during a pandemic [8,17,18,19,20,21,22,23,24].

Through the theoretical background and our own research, we have tried to identify, categorize, and evaluate the possibilities for using social media in online education during the pandemic from the perspective of selected teachers (qualitative research phase) and students of secondary schools in Slovakia (quantitative research phase). Teachers’ views on currently possible, as well as attractive, forms of online education and learning activities (which were implemented during the pandemic) seem to correspond to a large extent to the desired use of social media in education from the students’ point of view. This is evidenced by the most positively evaluated forms of online education (i.e., mainly four of the six categories).

The involvement of credible social media in education, which could lead to a mitigation of the negative effects of the pandemic on high school students, based on research findings in our research sample, positively affects *collaborative online education* in particular, which is based on group interactivity. This consists of at least two equally important processes: (a) Information building and (b) group collaboration of students who, in retrospect, build information (their opinions, values, or attitudes) based on group interactions. We can see that the two processes are interconnected and inviting, and make online education a social activity—even during a difficult pandemic. Students consider *attractive self-education* using social media (constructivism), *education using social networks* and education that uses social media as tools for *building relationships*.

This is confirmed by the research findings in our research sample, which were based on the evaluation of educational activities (i.e., research variables) that were actually implemented in Slovak secondary schools at the time of the pandemic. *Online lectures and courses within the YouTube channel* (Collaborative Education) dominated among the most positively evaluated variables, which also included *education with an emphasis on practical learning, i.e., learning by doing* (Active self-education); *project teaching—work in pairs or smaller groups* (Collaborative education); *obtaining information from educational institutions within the social network Facebook* (Education using social networks); and *indoor activities in the context of experiential learning* (Education with an emphasis on relationship behavior).

The intersection of the two monitored areas presents a picture of attractive possibilities and opportunities for online education (i.e., from the teachers’ view) and at the same time an idea of the desired uses of credible social media as tools contributing to maintaining interest in online education (i.e., the views and evaluation of the investigated high school students). To the above-mentioned intersection of teachers‘ and students’ views, the surveyed high school students would welcome online education during pandemics with a *greater degree of individual approaches to students* and a *greater involvement of smartphone educational applications in online education.*

Based on our research findings, we believe that a *greater degree of teacher individual approaches to students* (i.e., the first desired area identified among students) could bring a number of benefits, even in online education during a pandemic. In the questionnaire, for example, students stated that “personal conversation”, “student communication with the teacher without ‘witnesses´ (i.e., classmates)”, but also “solving problems directly with the student without the teacher writing to the parents” are welcome. Moreover, from a public health perspective, we might point out another observation; through the rich possibilities of social media, the individual approach of a teacher to a student can even, in a virtual space, contribute to the (perhaps unexpected) formation of students´ critical thinking [88], their better understanding of different dangers arising from social media [89], their views and acceptance of their own personal religion [90,91], multicultural or multireligious education [92], or thinking about own cultural values [93,94]. Similarly, students can also contribute to the formation of their own sustainable education [95], opportunities for improving learning [96], personal progress in technical skills [97], or skills in detecting manipulative elements of the media [89,98] under the conditions of the pandemic. In addition, an honest individual approach (for example through online personal interviews) can also raise necessary questions from the student about his/her own formation of his/her personal attitudes in relation to risky behaviors in the school environment [99], or even social radicalism and social fundamentalism [100]—including answers to complex questions such as the importance attributed by students to human authenticity [101], human roles and responsibilities [102], the quality of human life [103], or the quality of the environment [104], as well as to questions about the importance of human thinking and decision making [105] or about the meaning of one’s own beliefs [106,107].

Similarly, a *greater involvement of smartphone educational applications in online education* (i.e., the second most desired area identified by students) could be a good solution for alleviating or preventing many negative effects of the pandemic among student. The involvement of attractive smartphone educational applications in online education can be a personal motivator, for example, for fulfilling academic tasks [108], which has a positive impact on their frequent use or their long-term sustainability in education [8].

Strengthening the educational activities that our research has highlighted the most can bring fundamental challenges—not only for teachers, but also for creators of educational concepts and methodologies, for creators of educational policy agendas, or governments. There are similar challenges from the perspective of public health. We remain convinced that the involvement of credible social media and the use of their rich opportunities for current online education could be a welcome help during the pandemic, as well as helping to mitigate the negative effects of the pandemic on the younger generation, and thus also be helpful from the perspective of public health.

Based on the research findings, it can be stated that credible social media and their involvement in online education during a pandemic:

(1) Really change the way teachers interact with students;
(2) Help to involve students in the educational process and thus contribute to mitigating different negative effects of the pandemic;
(3) Change the status of students from often passive contributors (sitting on benches) to co-workers (sharing their ideas and results with classmates and students from other countries);
(4) Help teachers to link formal education with information that interests students, with attractive forms of knowledge presentation, and real-world things;
(5) Help teachers to implement forms of online education in which individual computer-based learning can also be understood as a social activity and act as elements for reducing the negative consequences of the COVID-19 pandemic.

Finally, we would like to speak about the research limitations that might be simultaneously considered as suggestions for future research. First of all, future research should use larger samples, as the sample in our research did not reflect the general population. At first it might be helpful to have a bigger number of research groups of students from a population.

Another limitation of this research may be the type of sampling of participants used, here performed by single-stage clustered sampling. In our opinion, multistage cluster sampling would be better, rather than collecting data from every single unit in the selected clusters. It might be effective to randomly select individual units from within the cluster to use as a research sample (i.e., two-stage sampling) as well as to take progressively smaller and smaller random samples (i.e., multistage sampling).

In the context of the tools used to collect this data, the previous validation study could have preceded the questionnaire. This would suitably supplement the already implemented trial phase (in the process of creating and formulating the questions), during which the selected students had to express whether the questionnaire was understandable enough to them. Moreover, a previous validation study could be helpful for researchers during verification of the accuracy of the measurements.

Ultimately, it would also be very welcome to separately compare the efficacy of concrete social media instruments (such as social sites, smartphones, etc.).

We certainly agree with the experts that there is already a need to consider proposals on “tailor-made interventions” that would be aimed at improving the health care, health, and quality of life of the populations most affected by the COVID-19 pandemic [108].

Moreover, as recent research suggests that young adolescents are the most vulnerable group in the pandemic [1,2,3,4,11,12,13,21,22,23,24,25], specific “toxic stressors” (such as family financial instability or disconnection from friends) should also be taken into account; these “stressors” [24,25,26] should be taken as other suggestions for future research that certainly affect the younger generation and its health.

Based on our research findings, some practical implications and suggestions given for future research might inspire consideration of the use of social media as a tool that might help improve education and current forms of online education, as well as lead students to experience a potential decrease in the negative effects of the pandemic [68,69,70]. This kind of research seems to be very significant because, as experts have pointed out, learning losses did not systematically increase, for example, with individual disease or as a consequence of other health or economic impacts of COVID-19—but were, in fact, the outcome of remote learning [5,6,23,109,110]. Therefore, it is important to ask, how meaningful has the role of social media within online education been so far [46,48,49,50,51,52,53,54,55], or how will the current pandemic affect the practice of social media education in the future?

The practical implications based on the findings of this study also bring another, final suggestion for future research. The careful consideration of long-term solutions that address the inequities in education that are exposed when schools are not physically accessible appear to be very important, including collaborative partnerships across sectors, in which teachers´ opinions will be a major contributor to any adopted solutions [23,111]. In this regard, we also consider an area of research that would focus on educational opportunities to be important, in which future education with a greater focus on students would be a priority.

## 6. Conclusions

To claim that the rich social media opportunities that are fast, easy, cheap, and available to most young people today and at any time are only helpful to young people during the coronavirus crisis would be misleading. Or, in the words of Ziming, many people rely on online content, but it is not entirely certain whether that content meets users’ expectations and needs [112]. The credibility and positive use of credible social media in education is therefore also proving to be an urgent area of research.

This article conducted an analysis of social media formulas applied to online education during the COVID-19 pandemic. The aim of this study was to (1) identify forms of online education (six categories) and educational activities (24 variables) and point out the specific possibilities of using social media in online education during a pandemic. As the activities actually carried out at the surveyed Slovak secondary schools were examined, we hope that the benefit of this research is not only the identification but also (2) the evaluation of forms of online education and educational activities that could contribute to mitigating the negative consequences of the pandemic on the younger generation. Ultimately, the presented results can also serve to support decision making on further measures and steps leading to the involvement of credible social media into the teaching methodologies now and in the future.

Our own research confirms that the opinions of research secondary school teachers (*n* = 9) and students (*n* = 102) are united, especially in view of the four currently possible (i.e., Possible during a pandemic) and at the same time attractive forms of online education with the involvement of credible social media. They are: (1) collaborative education; (2) active self-education—constructivism; (3) education using social networks; and (4) education with an emphasis on relationship behavior. At the same time, students rated the following learning activities (variables) as the most attractive: Online lectures and courses within the YouTube channel (Collaborative Learning); education with an emphasis on practical learning, i.e., learning by doing (Active self-education); project teaching—work in pairs or smaller groups (Collaborative education); gathering information from educational institutions within the social network Facebook (Education using social networks); and indoor experiential learning (Education with an emphasis on relationship behavior).

In the context of the research findings, it can be seen that social media, despite isolation during the pandemic, are proving to be popular tools for acquiring practical skills, more successful memorization of knowledge, and the more willing application of theoretical and practical knowledge and skills in real life. The current younger generation will use the acquired knowledge and skills not only in the period after the pandemic, but also later, after leaving the school desks. Accordingly, one can agree with experts who say that social media and their technological possibilities appear to be valuable resources in helping individuals cope with the current, and even also with longer-term, difficulties raised by the pandemic [113].

## Figures and Tables

**Table 1:** Currently possible as well as attractive uses of credible social media in online education—the view of teachers.

Identification of Forms of Online Education During the Pandemic (Research Categories)	Identification of Educational Activities (Research Variables)/Number of Teachers who Agreed (*n* = 9, in %)
Information education	(1) Poster creation (88.8%) (2) Production of video presentations and short films (100%) (3) Creation of thought maps (77.8%) (4) Creation of cartoon collages and comics (88.8%)
Collaborative education	(1) Interactive online quizzes (100%) (2) Online lectures and courses from partner educational institutions (100%) (3) Online lectures and courses on YouTube (88.8%) (4) Project teaching (work in pairs or smaller groups; 100%)
Education aimed at developing technological skills	(1) Information retrieval (77.8%) (2) Use of communication tools (88.8%) (3) Working with productive online tools (88.8%) (4) Working with graphic online tools (100%)
Education using social networks	(1) Information coming from educational institutions (Facebook; 100%) (2) Messenger as a communication channel for experiential learning activities and group projects (Facebook; 100%) (3) Student’s own research via questionnaire (Twitter; 88.8%) (4) Official websites of cultural institutions (100%)
Active self-education (constructivism)	(1) Project teaching (individual; 88.8%) (2) Education using podcasts (100%) (3) Education using brainstorming, group reflection (100%) (4) Education with an emphasis on practical learning (i.e., learning by doing; 100%)
Education with an emphasis on relationship behavior	(1) Community education and social assistance (100%) (2) Experiential learning (indoor activities; 88.8%) (3) Online workshops (88.8%) (4) Synchronous (online) communication (100%)

**Table 2:** Desired use and involvement of credible social media in online education—view and evaluation of students.

Forms of Online Education during a Pandemic	Maximum Number of Students Agreeing with the Value “I Find Attractive” (*n* = 102, in %)
Collaborative education	89.18%
Active self-education—constructivism	87.22%
Education using social networks	76.44%
Education with emphasis on relationship behavior	74.44%
Information education	10.78%
Education aimed at developing technological skills	7.84%
Individual approach of teachers to students	39.2%
Greater involvement of smartphone learning applications	33.32%

**Table 3:** The intersection of research findings—opinions of teachers and opinions of students.

Forms of Online Education during a Pandemic (Research Categories)	Educational Activities (Research Variables)	Maximum Number of Students Agreeing with the Value “I Find Attractive” (*n* = 102, in %)
Collaborative education	Online lectures and courses within the YouTube channel	34.3%
	Project teaching (working in pairs or small groups)	31.36%
	Interactive online quizzes	12.74%
	Online lectures and courses of partner educational institutions	10.78%
Active self-education (constructivism)	Learning by doing	32.34%
	Brainstorming and group reflection education	20.58%
	Podcasting	17.64%
	Project teaching (individual)	16.66%
Education using social networks	Information from educational institutions (Facebook)	29.4%
	Official websites of cultural institutions	20.58%
	Messenger as a communication channel for experiential learning activities and group projects (Facebook)	18.62%
	Own student research through a questionnaire (Twitter)	7.84%
Education with emphasis on relationship behavior	Experiential learning (within indoor activities)	25.48%
	Community education and social assistance	17.64%
	Synchronous (online) communication	16.66%
	Online workshops	14.7%
Information education	Creation of cartoon collages and comics	5.88%
	Poster design	1.96%
	Creation of thought maps	1.96%
	Production of video presentations and short films	0.98%
Education aimed at developing technological skills	Working with productive online tools	3.92%
	Use of communication tools	1.96%
	Information retrieval	0.98%
	Working with graphic online tools	0.98%

## Data Availability

The data presented in this study are available from the authors upon request.
